# Ideological differences in COVID-19 vaccine intention: the effects of trust in the healthcare system, in complementary and alternative medicine, and perceived threat from the disease

**DOI:** 10.3389/fpsyg.2024.1332697

**Published:** 2024-01-30

**Authors:** Monika Lamot, Katja Kerman, Andrej Kirbiš

**Affiliations:** ^1^Department of Sociology, Faculty of Arts, University of Maribor, Maribor, Slovenia; ^2^Department of Psychology, Faculty of Arts, University of Maribor, Maribor, Slovenia

**Keywords:** vaccine hesitancy, political ideology, perceived threat, trust in the healthcare system, complementary and alternative medicine

## Abstract

**Introduction:**

Politically left-leaning individuals are more likely to get vaccinated against COVID-19, although little is known about the mechanisms underlying the ideological differences in vaccination intentions. Understanding the extent to which trust in the healthcare system, in complementary and alternative medicine, and the perceived threat from the disease contribute to these disparities is crucial, as it could inform targeted interventions to address vaccine hesitancy across the political spectrum.

**Methods:**

The present cross-sectional study conducted among adults living in Slovenia (*n* = 858) examined the mediating role of trust in the healthcare system, trust in complementary and alternative medicine (CAM), and the perceived threat from the virus on COVID-19 vaccination intention.

**Results:**

We found that leftist ideology and trust in the healthcare system positively predicted vaccination intention, whereas CAM negatively predicted this intention. In addition, left-leaning individuals expressed greater trust in the healthcare system and lower trust in CAM, resulting in higher levels of COVID-19 vaccination intention. The serial mediation model confirmed that trust in CAM was a negative predictor, while trust in the healthcare system positively predicted perceived threat.

**Discussion:**

When dealing with vaccine hesitancy among right-oriented individuals, strategies should focus on enhancing trust in the healthcare system and critically evaluating the reliance on CAM.

## 1 Introduction

Considering the relatively large proportion of vaccine hesitant individuals in EU countries (European Commission, 2021), the COVID-19 crisis highlighted the need to better understand which groups of people may be more vaccine hesitant, and to gain insight into the mechanisms behind vaccine hesitancy. Although previous literature has identified political ideology as a relevant factor in vaccine hesitancy (Callaghan et al., 2021; Latkin et al., 2021), comprehensive and fine-grained investigations into the underlying mechanisms—specifically, the role of trust in the healthcare system, in complementary and alternative medicine (CAM), and the perceived threat from the disease—are absent. This study aims to fill this critical literature gap, offering insights that could be pivotal for developing nuanced public health strategies tailored to varying political beliefs. Specifically, we build on previous literature (Ledford et al., 2022) to examine the mediating role of trust in the healthcare system and complementary and alternative medicine in the relationship between political ideology and vaccine intention. Furthermore, we incorporated an assessment of perceived threat into our analysis. This addition is supported by recent research from Fleury-Bahi et al. (2023), which highlights the importance of perceived risk and institutional trust in explaining individuals’ vaccination intention.

### 1.1 Ideological differences in vaccination intention

The link between political orientation and vaccine intention has been well established in the literature, with studies consistently showing that the politically right-oriented public (i.e., conservatives) expresses lower COVID-19 vaccine intention uptake and lower vaccine trust (Ward et al., 2020; Latkin et al., 2021). For example, a study from the United States has shown that in a multivariate model comparing over 20 predictors, conservative political ideology was the second strongest predictor of vaccine intention uptake (Rosenfeld and Tomiyama, 2021). However, there is a lack of research, comprehensively investigating mechanisms that may play a role in the relationship between political ideology and COVID-19 vaccine intention.

### 1.2 Explaining the link between political differences in COVID-19 vaccination intention through trust in the healthcare system, in CAM and perceived threat from the disease

Prior research suggests that perceived threat is among the key mechanisms explaining the link between liberal/left political ideology and greater vaccine intention uptake. The perceived threat consists of perceived susceptibility and perceived severity of the disease (Champion and Sugg Skinner, 2008; Vrdelja et al., 2020). Studies prior to the COVID-19 pandemic have shown that the perceived threat from a vaccine-preventable disease is an important predictor of vaccine acceptance (Panchapakesan et al., 2018; Chang and Lee, 2019). In line with these findings, studies have shown that COVID-19 vaccine acceptance is predicted by both higher perceived susceptibility (Guidry et al., 2020) and perceived severity (Qiao et al., 2020). While higher perceived COVID-19 threat is associated with greater vaccine intention (Dodd et al., 2021; Lackner and Wang, 2021), and is typically higher among left-oriented individuals (Calvillo et al., 2020; Fridman et al., 2021), the lower perception of threat from COVID-19 among conservatives in the majority of studies is inconsistent with research prior to the pandemic, which consistently showed that conservatives/rightists expressed higher perceived threat from the infectious diseases (Jost et al., 2003; Conway et al., 2020; Pavlovic et al., 2021). This apparent contradiction may be explained by the politicization of the pandemic, which has potentially altered the typical conservative attitude of risk-aversion into a politicized assessment of the COVID-19 threat, an influence that overrides prior ideological tendencies toward threat perception (Peretti-Watel et al., 2020; Galvão, 2021). Taken together, we rely on previous literature and findings, and propose that the threat from COVID-19 plays a mediating role in the relationship between leftist ideology and vaccine intention.

*Hypothesis 1:* The relationship between leftist ideology and vaccination intention is mediated by the perceived threat from COVID-19; i.e., individuals with a leftist ideology are likely to perceive a higher threat of the disease, which increases vaccination intention.

Trust in the healthcare system has been linked to positive vaccine attitudes (Browne et al., 2015; Baumgaertner et al., 2018), yet fewer studies have examined how trust in the healthcare system varies by political ideology. Conservatives often exhibit skepticism toward science (Kossowska et al., 2021) and diminished trust in information provided by medical professionals (Motta et al., 2018). Political ideology may affect what information is selected and deemed trustworthy so that it aligns with one’s political beliefs (Motta et al., 2018). This selective trust based on ideology likely extends to the healthcare domain, where it can influence attitudes toward vaccination. Liberal individuals may have greater trust in science because such attitudes are more in line with the values and beliefs of democratic, leftist parties (Joslyn and Sylvester, 2019), which could translate into greater acceptance of health interventions endorsed by scientific consensus, such as vaccines. Conversely, conservatives’ skepticism toward science may decrease trust in both medical professionals and public health institutions like the CDC in the United States (Baumgaertner et al., 2018), potentially contributing to a reliance on personal judgment or alternative sources of health information, such as CAM. Given left-oriented individuals’ greater trust in medical professionals and science, we expect them to express higher trust in healthcare institutions.

*Hypothesis 2:* The relationship between leftist ideology and COVID-19 vaccination intention is mediated by trust in the healthcare system. In other words, individuals with a leftist ideology are more likely to express greater trust in the healthcare system, which increases vaccination intention.

In contrast to trust in the healthcare system, endorsement of complementary and alternative medicine (CAM) negatively predicts pro-vaccine attitudes (Rozbroj et al., 2019; Hornsey et al., 2020b). Those endorsing CAM may perceive conventional medicine as untrustworthy (Hornsey et al., 2020b). We argue that it is necessary to examine the role of political ideology in CAM endorsement. Specifically, we propose that leftists are less likely to trust CAM since they express greater trust in the healthcare system (Baumgaertner et al., 2018). Additional corroboration of the link between political ideology and endorsement of CAM arises from the association of CAM with “pseudo-reliance bullshit,” which Frankfurt (2005) describes as a communication style aimed at persuading the audience without concern for the truth, evidence, or accepted knowledge. Alternative medicine falls under “pseudo-profound reliance” because it does not necessarily support empirical evidence (e.g., the importance of vaccinations; Petrocelli, 2021). In addition, receptiveness to reliance on pseudo information has previously been associated with alternative medicine endorsement (Pennycook et al., 2015; Čavojová et al., 2019) and a right-leaning political orientation (Pfattheicher and Schindler, 2016; Sterling et al., 2016; Nilsson et al., 2019). Although research directly linking political ideology and CAM is scarce, leftists’ lower receptivity to pseudo-information and greater trust in healthcare suggest they are less likely to endorse CAM.

*Hypothesis 3:* The relationship between leftist ideology and vaccination intention is mediated by trust in CAM, i.e., left-oriented individuals are less likely to trust CAM, which would increase vaccination intention.

### 1.3 Does trust in the healthcare system and in CAM relate to an individual’s perception of threat from the disease?

Previous studies show that trust in CAM negatively predicts vaccine hesitancy (Rozbroj et al., 2019; Hornsey et al., 2020b), whereas the opposite was found for trust in the healthcare system (Baumgaertner et al., 2018; Justwan et al., 2019). We propose that the relationship between trust in CAM and trust in the healthcare system is further mediated by the perceived threat/risk from COVID-19, a significant predictor of COVID-19 vaccination intention (Guidry et al., 2020; Qiao et al., 2020). Studies during the COVID-19 pandemic have shown that obtaining information from credible sources, such as the WHO (Karasneh et al., 2021), and trust in healthcare professionals contribute to a higher perceived risk from the disease (Pickles et al., 2021; Schneider et al., 2021). A study in Europe, Asia, and America confirmed that trust in medical professionals impacts the perceived threat from COVID-19 disease (Dryhurst et al., 2020), suggesting a positive association between trust in the healthcare system and perceived threat from the disease. Although research, examining the link between CAM and the perceived threat from COVID-19 is scarce, a Norwegian study conducted among CAM providers found that more than half of respondents expressed no concern about contracting COVID-19 (Stub et al., 2021), which could suggest that CAM is negatively associated with perceived threat.

*Hypothesis 4:* The relationship between leftist ideology and vaccination intention is serially mediated via trust in the healthcare system and perceived threat. In other words, leftist ideology increases trust in the healthcare system, which increases perceptions of threat from the disease, which in turn increases vaccination intention.

*Hypothesis 5:* The relationship between left ideology and vaccination intention is serially mediated via CAM and perceived threat, i.e., leftist ideology decreases trust in CAM, which increases perceived threat of the disease, which in turn increases vaccination intention.

## 2 Methods

### 2.1 Procedure

The cross-sectional study was conducted between March and April 2020 during Slovenia’s first lockdown. The study was promoted via social media and a nonprobability sample was collected online, using the 1 ka platform.^1^ Participants were first given an overview of the study, including its aims and objectives. They were informed that participation was completely anonymous and voluntary, and that they could opt out at any time. All participants provided informed consent prior to participation.

### 2.2 Participants

The sample consisted of 858 respondents from Slovenia (84.6% female and 15.4% male). Participants were between 18 and 69 years old (*M* = 33.6). 68.3% reported being employed, 23.1% were students, 7.3% were unemployed, 0.9% were retired, and 0.4% were attending high school. The sample consisted mostly of highly educated participants; 34.9% reported having a bachelor’s degree, 28.1% had a master’s degree, and 5.4% reported having a PhD. Additionally, the average political orientation score in the sample was 5.42, indicating a central tendency in political views among the participants.

### 2.3 Materials

#### 2.3.1 Perceived threat from COVID-19

Perceived threat from COVID-19 was assessed with perceived susceptibility and perceived severity, two dimensions of perceived threat (Champion and Sugg Skinner, 2008). Susceptibility was measured with the item “How likely do you believe it is that you could get COVID-19?” with answer options ranging from 0 (not at all likely) to 10 (very likely). Perceived severity was assessed with the item “How dangerous/threatening do you believe COVID-19 is for one’s health?” with answer options ranging from 1 (not threatening at all) to 5 (very threatening). Following the literature on measuring perceived threat (e.g., Weinstein, 2000; Skinner et al., 2015; Chen and Liu, 2021), we multiplied the items of perceived susceptibility and severity. The correlation between the two items was strong (ρ = 0.78; *p* < 0.01).

#### 2.3.2 Trust in the healthcare system

To measure trust in the healthcare system, we adapted the Revised Health Care System Distrust Scale (Shea et al., 2008) to the context of the Slovenian health care system. The following three items (“The healthcare system in Slovenia does its best to make patients’ health better,” “The healthcare system in Slovenia covers up its mistakes,” and “The healthcare system in Slovenia puts making money above patients’ needs”) were assessed on a five-point Likert scale (1 = completely disagree; 5 = completely agree). The scores on the last two items were reverse coded so that higher scores on all items indicate greater trust in the healthcare system. A single composite score was computed, based on the three indicators by calculating the mean score. The scale showed adequate reliability (α = 0.85).

#### 2.3.3 Complementary and alternative medicine

Two items were developed to measure attitudes toward complementary and alternative medicine (CAM) for the purposes of this study (“Complementary and alternative medicine include natural herbal formulas that are healthier than medications prescribed by a doctor” and “Complementary and alternative medicine are generally a better way to treat a disease”). Both items were measured on a five-point Likert scale (1 = completely disagree; 5 = completely agree). A composite variable, based on calculating the mean score was used to examine attitudes toward CAM. The items correlate strongly (ρ = 0.83; *p* < 0.01).

#### 2.3.4 Political ideology

To assess political ideology, we used the standard single item (ESS, 2022). Specifically, we asked the respondents: “In politics people sometimes talk of ‘left’ and ‘right’. Where would you place yourself on this scale, where 1 means the left and 11 means the right?”

#### 2.3.5 COVID-19 vaccination intention

Vaccination intention was measured with a single self-developed item: “What is the probability that you would get vaccinated against the coronavirus if the vaccine were available?” with responses ranging from 1 (unlikely at all) to 11 (very likely).

#### 2.3.6 Control variables

Three control variables were included in the model: gender (1 = male, 2 = female), age (in years), and education (1 = primary education or less, 2 = lower or secondary vocational education, 3 = secondary technical education, 4 = gymnasium, 5 = Bachelor’s degree, 6 = Master’s degree, 7 = PhD).

### 2.4 Statistical analyses

We tested the hypotheses in a single structural equation model in Mplus 8, using the maximum likelihood estimator. In addition to the hypothesized direct effects, correlation terms were added between trust in the health care system and CAM, resulting in a saturated model. We estimated the indirect effects in R, using the Monte Carlo confidence intervals (Preacher and Selig, 2012). Preliminary analyses were carried out in Mplus 8.3 to examine the factor structure of the scales used in the study.

## 3 Results

Descriptive statistics (*M* and *SD*) and correlations between variables are presented in Table 1. Bivariate correlations revealed a statistically significant negative correlation between political orientation and vaccination intention (*r* = −0.23, *p* < 0.01), as well as between political orientation and perceived threat (*r* = −0.13, *p* < 0.01) and between political orientation and trust in the healthcare system (*r* = −0.16, *p* < 0.01). Additionally, there was a positive, statistically significant correlation between political orientation and CAM (*r* = 0.26, *p* < 0.01).

**Table 1:** Descriptive statistics and bivariate correlations.

	*N*	*M*	*SD*	PI	VI	TH	HS	CAM
PI	838	5.42	2.08	-				
VI	858	4.69	4.25	−0.23^**^	-			
TH	857	18.11	16.68	−0.13^**^	0.78^**^	-		
HS	858	2.70	0.99	−0.16^**^	0.60^**^	0.52^**^	-	
CAM	858	3.33	1.19	0.26^**^	−0.69^**^	−0.53^**^	−0.63^**^	-

PI, Political orientation; VI, Vaccination intention; TH, Perceived threat; HS, Trust in the healthcare system; and CAM, Complementary and alternative medicine. ^**^p < 0.01.

A single confirmatory factor model was tested, with items loading on their respective factors. Confirmatory factor analysis showed an acceptable to good model fit to the data (χ^2^ = 39.411; df = 11; RMSEA = 0.06; CFI = 0.99; SRMR = 0.02; Hu and Bentler, 1999), and all items load highly (> 0.70) to their respective factors.

### 3.1 Direct and indirect effects

**Figure 1:**
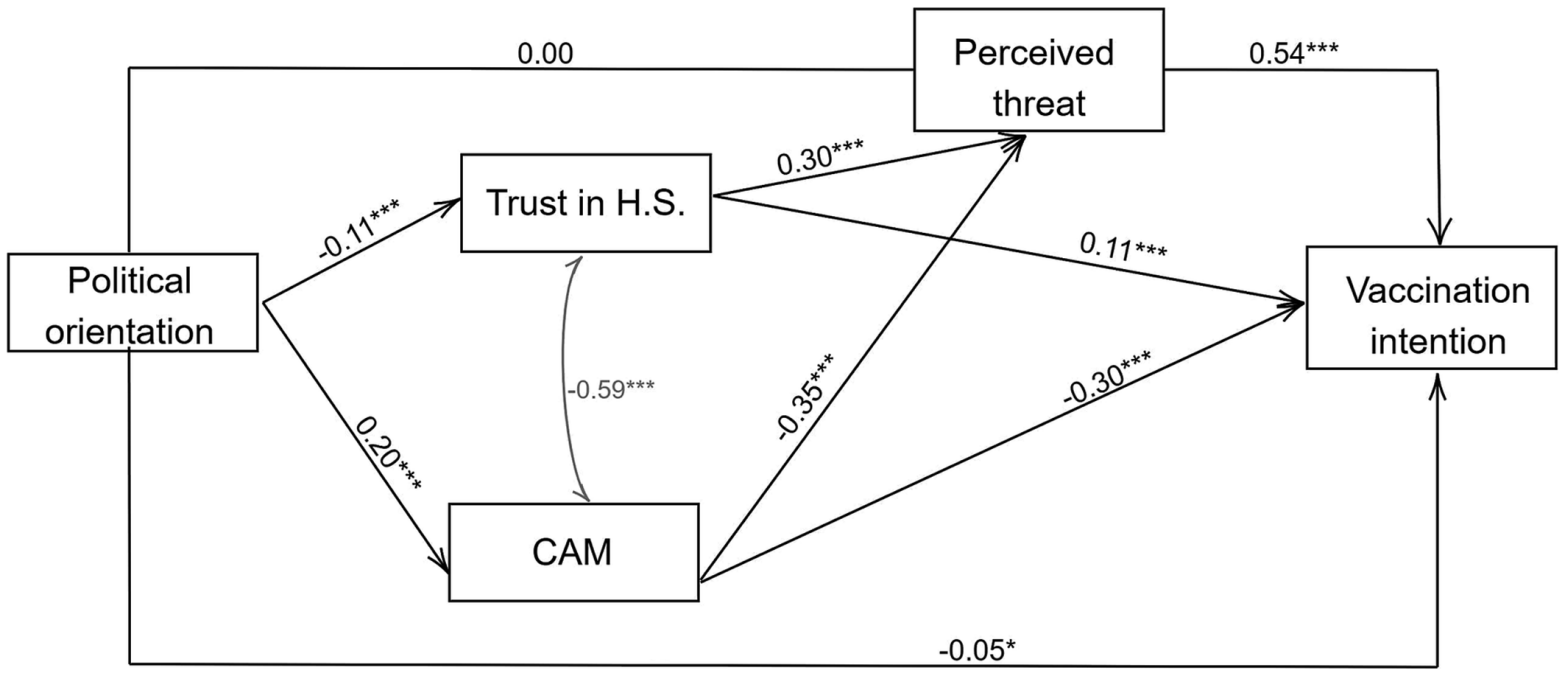
Estimation of the direct effects. *R*^2^ = 0.74. Reported are the standardized estimates and unstandardized *p* values. ^*^p < 0.05, ^**^p < 0.01, and ^***^p < 0.001.

The results of mediation analysis are displayed in Figure 1. In this model, we integrated the demographic control variables—gender, age, and education—directly into the analysis to account for their potential influence on the primary relationships examined. Political orientation significantly and positively predicted trust in CAM (*b* = 0.20, *p* < 0.001), and significantly negatively predicted trust in the healthcare system (*b* = −0.11, *p* < 0.01). Furthermore, trust in the healthcare system (*b* = 0.11, *p* < 0.001) and perceived threat (*b* = 0.54, *p* < 0.001) significantly and positively predicted vaccination intention, while trust in CAM negatively predicted vaccination intention (*b* = −0.29, *p* < 0.001). In addition, trust in the healthcare system significantly and positively predicted perceived threat (*b* = 0.30, *p* < 0.001), while trust in CAM significantly and negatively predicted perceived threat (*b* = −0.35, *p* < 0.001).

With regard to control variables (Supplementary Table S1), we found that older individuals were less likely to get vaccinated (*b* = −0.13, *p* < 0.001), were more likely to trust in CAM (*b* = 0.27, *p* < 0.001), and were less likely to trust the healthcare system (*b* = −0.20, *p* < 0.001). Age, however, was not a significant predictor of the perceived threat from COVID-19. We also found that women were more likely to perceive COVID-19 as threatening (*b* = 0.10, *p* < 0.01), held more positive attitudes toward CAM (*b* = 0.16, *p* < 0.001), expressed less trust in the healthcare system (*b* = −0.11, *p* < 0.001), and showed lower vaccination intention (*b* = −0.05, *p* < 0.01). In addition, our findings revealed that education did not predict vaccination intention; however, more educated individuals expressed lower perceived threat (*b* = −0.08, *p* < 0.05), were less likely to trust CAM (*b* = −0.12, *p* < 0.001), and were more likely to trust the healthcare system (*b* = 0.12, *p* < 0.01).

The Monte Carlo analysis displayed in Table 2 revealed several statistically significant parallel indirect effects. Specifically, the confidence intervals showed a significant relationship between leftist ideology and vaccine intention via CAM and via trust in the healthcare system, confirming Hypotheses 2 and 3, respectively. We did not find a significant indirect effect between political ideology and vaccine intention via perceived threat, leading us to reject Hypothesis 1. Regarding the serial mediation analysis, the results revealed that the examined indirect effects were significant. In other words, a significant indirect effect was observed between leftist ideology and vaccine intention via CAM and trust in the healthcare system, followed by perceived threat. Thus, the results supported Hypotheses 4 and 5.

**Table 2:** Monte Carlo analysis of indirect effects.

		Monte Carlo C.I.	
	Est.	LL	UL
Left ideology ➔ CAM ➔ Vaccine intention	−0.109	**−0.1513**	**−0.071**
Left ideology ➔ Trust in the health care system ➔ Vaccine intention	−0.021	**−0.040**	**−0.006**
Left ideology ➔ Perceived threat ➔ Vaccine intention	0.000	−0.059	0.060
Left ideology ➔ CAM ➔ Perceived threat ➔ Vaccine intention	−0.139	**−0.198**	**−0.087**
Left ideology ➔ Trust in the health care system ➔ Perceived threat ➔ Vaccine intention	−0.064	**−0.112**	**−0.022**

95% confidence intervals (C.I.) are provided for each estimate. Significant confidence intervals are shown in bold.

## 4 Discussion

In our study, we examined potential mechanisms in the relationship between political ideology and vaccination intention. The results revealed several important findings. First, we found that leftist ideology and trust in the healthcare system positively predicted COVID-19 vaccination intention, whereas trust in CAM negatively predicted the intention to get vaccinated, consistent with previous research (e.g., Justwan et al., 2019; Ward et al., 2020; Hornsey et al., 2020a). However, the results showed a nonsignificant relationship between an individual’s political orientation and perceived threat. This is a departure from prior literature, which has generally found conservative individuals to exhibit a greater perceived threat from infectious diseases (Jost et al., 2003). It is possible that the unique context of the COVID-19 pandemic, which has been highly politicized (Peretti-Watel et al., 2020; Galvão, 2021), may have influenced traditional patterns of risk perception. The findings thus suggest that the relationship between political orientation and perceived threat may be more complex and context-dependent, which warrants further investigation in future studies.

Based on parallel mediation, the results confirmed that trust in the healthcare system and CAM mediate the relationship between political orientation and vaccination intention. In other words, individuals with leftist political views expressed greater trust in the healthcare system and lower trust in CAM, resulting in higher COVID-19 vaccination intention. These findings are consistent with pre-COVID-19 research showing that leftists have more trust in medical professionals and science (e.g., Baumgaertner et al., 2018). Greater trust in the healthcare system could also explain distrust of CAM (see, for example, Browne et al., 2015). However, we found no evidence for the mediating role of perceived threat in the relationship between political orientation and vaccine intention.

Furthermore, we were interested in examining how trust in the healthcare system and in CAM relate to vaccination intention. Serial mediation confirmed the significant role of perceived threat. Specifically, the results showed that trust in CAM negatively predicted perceived threat, whereas the opposite was true for trust in the health care system. Leftists are more likely to trust the healthcare system, which positively affects perceived threat and leads to higher vaccination intention. In addition, leftists are less likely to have confidence in CAM, which increases perceived threat (considering that CAM negatively predicts threat) and positively affects the intention to get vaccinated against COVID-19.

Our study has several implications. Most importantly, when communicating about vaccination, it is crucial to address the groups that express reservations about vaccines, while taking into account the mechanisms that underlie vaccine skepticism. Thus, since there are significant differences in vaccine intention and uptake between people with different ideological orientations (e.g., Callaghan et al., 2021; Kerr et al., 2021), public health communication should focus particularly on the underlying factors that contribute to such differences. For example, when dealing with vaccine hesitancy among right-oriented individuals, special consideration should be given to trust in CAM and the healthcare system. Because CAM is a significant predictor of vaccine hesitancy and right-oriented people are more likely to trust CAM and are therefore exposed to pseudo-CAM information, health communication should focus on debunking false statements from CAM providers, publications, the Internet, and social media websites. One possible way would be to advertise facts about vaccines on CAM online websites. However, some CAM practitioners might actively discourage vaccination, an attitude which could produce resistance to hosting such advertisements. To address this issue, a research-based approach to understanding CAM users’ motives and views is critical. Surveys or focus groups could be used to effectively customize communication and advertising methods. Finally, to ensure the most efficient strategy, it is critical to employ experimental studies examining the effectiveness of various communication strategies for vaccine hesitant, right leaning individuals. It should also be emphasized that the recommendation to focus communication strategies on debunking CAM misinformation among right-oriented individuals should not be misconstrued as overlooking the diversity of political beliefs within a population. We suggest a targeted approach within a comprehensive public health communication strategy that also considers the spectrum of ideological orientations. Future research could explore the efficacy of such communication approaches in politically diverse settings.

Although our study provides insight into the mechanisms of ideological difference in COVID-19 vaccination intention, several limitations of the study must be considered when interpreting the results. First, our sample is not representative, limiting the generalizability of our findings. Second, the results of direct and indirect effects must be interpreted with caution because of the study’s cross-sectional design. Thus, future studies should further examine trust in CAM and the healthcare system as mechanisms in the relationship between political ideology and vaccine intention with a longitudinal study design, which would enable the examination not only of direct relationships, but of reverse-causal ones as well.

## Data availability statement

The raw data supporting the conclusions of this article will be made available by the authors, without undue reservation.

## Ethics statement

In line with national and institutional guidelines, ethical approval is not required for low or no-risk cross-sectional studies. The research was conducted in accordance with the guidelines of the Helsinki Declaration and its later amendments. Participants were first given an overview of the study, including its aims and objectives. They were informed that participation was completely anonymous and voluntary, and that they could opt out at any time. All participants provided informed consent prior to participation.

## Author contributions

ML: Formal analysis, Writing – original draft, Writing – review & editing. KK: Formal analysis, Writing – review & editing. AK: Writing – original draft, Writing – review & editing.

## Footnotes

^1^ https://www.1ka.si
