# Supplementary material for: Ideological differences in COVID-19 vaccine intention: the effects of trust in the healthcare system, in complementary and alternative medicine, and perceived threat from the disease
Source: Front Psychol. 2024 Jan 30;15:1332697. doi: 10.3389/fpsyg.2024.1332697 (PMC10875337; doi:10.3389/fpsyg.2024.1332697)
Supplement: Supplementary file 1 [file Table_1.docx]

Supplementary Material

# Supplementary Data

Table S1. Effects of control variables.

|  | Trust in the HS | | CAM | | Perceived threat | | Vaccine intention | |
| --- | --- | --- | --- | --- | --- | --- | --- | --- |
|  | b (S.E.) | z | b (S.E.) | z | b (S.E.) | z | b (S.E.) | z |
| Gender | -0.11 (0.03)^***^ | -3.39 | 0.16 (0.04)^***^ | 4.30 | 0.10 (0.03)^***^ | 3.70 | -0.05 (0.02)^**^ | -3.00 |
| Age | -0.20 (0.04)^**^ | -5.80 | 0.27 (0.03)^***^ | 7.91 | -0.03 (0.04) | -0.83 | -0.13 (0.02)^***^ | -6,40 |
| Education | 0.12 (0.04)^***^ | 3.16 | -0.12 (0.04)^***^ | -3.31 | -0.08 (0.03)^*^ | -2.39 | 0.01 (0.02) | 0.64 |

*Note*. HS = healthcare system, CAM = complementary and alternative medicine. *** *p* < .001, ** *p* < .01.
